# Supplementary material for: Content-rich biological network constructed by mining PubMed abstracts
Source: BMC Bioinformatics. 2004 Oct 8;5:147. doi: 10.1186/1471-2105-5-147 (PMC528731; doi:10.1186/1471-2105-5-147)
Supplement: Additional File 5 — The original Chilibot query results of the term "long-term potentiation (LTP)" and 22 other terms, limiting the latest references analyzed to the years 1990, 1995, 2000, and 2004. [file 1471-2105-5-147-S5.bz2 › chilibotAdditionalFile5/ltp2000/html/LTP_ACTININ.html]

 


 **LTP** and **ACTININ** 
  
Found 1 abstracts in PubMed,  **1 abstracts were retrieved and analyzed**.  


---

 Search Google  |
 PDF files only 
|  EDU domain only 

---

- Eur J Pharmacol, 1999   The glutamatergic synapse is the key structure in the development of activity dependent synaptic plasticity in the central nervous system.
  The analysis of the complex biochemical mechanisms at the basis of the long term changes in synaptic efficacy have received a tremendous impulse by the observation that the post synaptic constituents of the synapse can be separated and purified through a simple procedure involving detergent treatment of synaptosomes and differential centrifugation.
  In this fraction, called post synaptic density , the functional interactions of its constituents are preserved.
  The various subunits of ionotropic glutamate receptors are held in register with the presynaptic active zone through their interaction with linker proteins.
  N methyl D aspartate subunits NR2A and NR2B, bind to the PSD protein called PSD 95, which in turn binds neuroligins, providing a handle for interacting with neurexin, located in the plasma membrane at the presynaptic active zone.
  Additional clustering of NMDA receptors is provided through the binding of NRI subunits to the cytoskeletal protein alpha **actinin** 2.
  AMPA and kainate receptors are other important constituents of PSDs and bind to different anchoring proteins.
  Phosphorylation processes have long been known to modulate NMDA receptor functional activity the finding that several protein kinases, particularly Ca2ion Calmodulin dependent protein kinase II and protein tyrosine kinases of the src family, are major constituents of PSDs has allowed to demonstrate that these enzymes are localized in a strategic position of the glutamatergic synapse, so that their activation provides a means for NMDA receptor function regulation upon its activation.
  The relevance of these mechanisms has been demonstrated in experimental models of pathologies involving deficits in synaptic plasticity, such as in streptozotocin induced diabetes and in an animal model of prenatal induced ablation of hippocampal neurons.
  Both animal models display disturbances in long term potentiation **[LTP]** and cognitive deficits, thus providing in vivo models to study pathology related changes in both the structure and the function of the excitatory synapse.
